# The Relationship Between Mindfulness, Depression, Anxiety, and Quality of Life in Individuals With Schizophrenia Spectrum Disorders

**DOI:** 10.3389/fpsyg.2021.708808

**Published:** 2021-08-31

**Authors:** Niklas Bergmann, Eric Hahn, Inge Hahne, Marco Zierhut, Thi Minh Tam Ta, Malek Bajbouj, Geradina Henrika Maria Pijnenborg, Kerem Böge

**Affiliations:** ^1^Department of Psychiatry and Psychotherapy, Campus Benjamin Franklin, Charité – Universitätsmedizin Berlin, a corporate member of Freie Universität Berlin, Humboldt-Universität zu Berlin, and Berlin Institute of Health, Berlin, Germany; ^2^Department Clinical Psychology and Experimental Psychology, Faculty of Behavioral and Social Sciences, University of Groningen, Groningen, Netherlands

**Keywords:** mindfulness, psychosis, schizophrenia spectrum disorders, depression, anxiety, quality of life, mediation, transdiagnostic

## Abstract

**Background:** Schizophrenia spectrum disorders (SSD) are frequently accompanied by comorbid depressive and anxiety symptoms, as well as impaired quality of life (QoL). A growing body of evidence has demonstrated the relevance of mindfulness for SSD in recent years. The study examined the association between mindfulness, depression, anxiety, and QoL.

**Materials and Methods:** A total of 83 participants with SSD were recruited at the in- and outpatient psychiatric hospital care. Participants completed the Southampton Mindfulness Questionnaire, Comprehensive Inventory for Mindful Experiences, and Freiburger Mindfulness Inventory, the Depression, Anxiety, Stress Scale to assess depression and anxiety, and the WHO-QoL Questionnaire. Multiple regression analyses examined the relationship between mindfulness and QoL and the mediating role of depression and anxiety.

**Results:** Mindfulness had a significant statistical positive effect on QoL domains physical health, psychological, and environmental QoL in patients with SSD. Depression was identified as a significant mediator of this relationship.

**Conclusion:** This study provides novel insight into mindfulness’ mechanisms and paves the way for a process-oriented approach to treat SSD. The results provide first evidence for the process-based value of mindfulness for SSD; future studies can focus on the role of mindfulness for central therapeutic processes of change by employing longitudinal designs.

## Introduction

Schizophrenia spectrum disorders (SSD) constitute severe neuropsychiatric conditions, affecting 1.1–1.8% of the general population (Global Burden of Disease Study Collaborators, 2015). In about two-thirds, relapses and continued impairments occur (Smith et al., 2010), and many individuals with SSD are diagnosed with comorbid disorders, such as depression or anxiety (Fusar-Poli et al., 2014; Moritz et al., 2019). Core features of SSD comprise positive and negative symptoms, cognitive dysfunctions (Krynicki et al., 2018), and affective symptoms (American Psychiatric Association, 2013).

Positive symptoms primarily consist of delusions, hallucinations, and disorganized thinking, whereas negative symptoms are frequently characterized by a loss or reduction of specific social functioning domains (Lincoln et al., 2017). Post-psychotic depression is highly prevalent in SSD, ranging between 20% in individuals with chronic SSD and 50% in individuals after receiving treatment for a first psychotic episode (Upthegrove et al., 2016). Furthermore, up to 80% experience a depressive episode during the early stages of SSD progression, resulting in a poorer prognosis, higher suicidality (Upthegrove et al., 2016), and decreased quality of life (QoL; Saarni et al., 2010).

A recent meta-analysis has highlighted the difficulties in validly distinguishing symptoms of depression from negative symptoms, suggesting a dimensional view of negative, positive, and depressive symptoms instead of categorical entities separating depression and negative symptoms (Krynicki et al., 2018). The distinction between depression and negative symptoms could be made based on the underlying origins of the symptoms: whereas low motivation is a driving cause in negative symptoms, reduced energy, and drive or low mood define depressive symptoms (Edwards et al., 2019). For instance, social withdrawal can be considered as *inherent* to negative symptoms as the individual suffers from a lack of social motivation and reward as a key driver to stay in contact with other people. In contrast, individuals with depression refrain from social contacts as a *consequence* of their low mood and low energy level (Edwards et al., 2019). Other central features of depression comprise low self-esteem, experiential avoidance (Udachina et al., 2014), rumination, and worry (Hartley et al., 2014).

Besides depressive symptoms, anxiety symptoms are very common in SSD and occur in about 65% of patients with schizophrenia. Around 38% fulfill the criteria for anxiety disorders, with some of the most common ones being social anxiety disorder (15%), generalized anxiety disorder (11%), and panic disorder (10%; Temmingh and Stein, 2015). Anxiety toward socially salient cues plays a central role in the development of persecutory delusions experienced by individuals with SSD (Achim et al., 2011). Cognitive symptoms, very prevalent in SSD, might impair the individuals’ ability to effectively use strategies to deal with general stressors in life but also the symptoms of SSD, resulting in maladaptive coping strategies. Despite the large relevance of anxiety in SSD, however, it remains one of the least examined features of the disorder (Buonocore et al., 2017).

Research found that anxiety in individuals with SSD is related to impaired daily functioning, lower self-esteem, elevated distress, relapse, and suicide rates (Buonocore et al., 2017). Furthermore, individuals with SSD and comorbid anxiety disorder report lower QoL compared to individuals with SSD without a comorbid anxiety disorder (Achim et al., 2011). Even when controlling for positive and negative symptoms as well as for depression, higher anxiety levels were found to be associated with reduced QoL (Temmingh and Stein, 2015). Despite this evidence, however, the causality of the relationship between anxiety and such clinical outcomes remains ambiguous, as it is unclear whether anxiety causes adverse clinical outcomes or vice versa (Buonocore et al., 2018). Interventions selectively targeting anxiety in individuals with SSD were associated with a more promising course of illness and clinical outcomes than interventions that did not target anxiety symptoms (Buonocore et al., 2017; McAusland et al., 2017).

In recent years, a growing body of literature has demonstrated the promising effects of mindfulness-based interventions (MBIs), aiming to help the patients to develop a mindful and accepting attitude toward their symptoms and distressing experiences (Jansen et al., 2020). Although there is no universally defined concept of mindfulness, it can be conceptualized as a state of consciousness involving a non-judgmental attitude, awareness of inner thoughts, emotions and sensations, and inner acceptance toward them. Practicing mindfulness therefore encompasses a mental state of non-judgment, nonreactivity, detachment, and acceptance and compassion toward oneself and others (Baer, 2003).

For individuals with SSD, mindfulness skills might help the individual to become aware of unusual experiences, such as hallucinations or delusions, and learn to cope with them in an accepting way (Chadwick, 2014). Consequently, mindfulness was correlated with lower depression and anxiety in individuals with SSD (Rayan, 2017). However, the relationship between mindfulness and anxiety is ambiguous, as a recent meta-analysis reports anxiety in SSD not to be improved by MBIs (Jansen et al., 2020). Additionally, mindfulness skills were found to be related to a reduction in affective and negative symptoms (Louise et al., 2018; Jansen et al., 2020) and an increase in functioning as well as QoL (Khoury et al., 2013; Jansen et al., 2020). More specifically, in a randomized-controlled trial, individuals in a MBI group compared to a control group were found to improve in scores of the WHO QoL questionnaire regarding psychological and environmental QoL as well as physical health, whereas social relationships were not affected (López-Navarro et al., 2015). The same results were indicated in a recent pilot trial examining the effects of mindfulness-based group therapy for SSD (Böge et al., 2021).

To summarize, research implies an association between mindfulness and improvements in anxiety, depression (Hofmann and Gómez, 2017), and QoL (Khoury et al., 2013; Rayan, 2017). Furthermore, independent of MBIs, it has been shown that interventions targeting anxiety and depression in individuals with SSD have potential benefits, including an increase in QoL (Buonocore et al., 2017), which is associated with a better chance of recovery (Achim et al., 2011). However, no study has examined the relationship between these factors in order to shed light on the probable mechanisms of depressive and anxiety symptoms underlying the effect of mindfulness on QoL in SSD.

This study examined the relationship between mindfulness, depression and anxiety, and QoL in individuals with SSD. More precisely, it was hypothesized that in individuals with SSD (H1) high levels of mindfulness are associated with high QoL. Furthermore, it was expected that (H2) high levels of mindfulness are associated with lower levels of depression and anxiety; and lastly, that (H3) depression and anxiety mediate the relationship between mindfulness and QoL.

## Materials and Methods

### Design and Procedure

**Figure 1:**
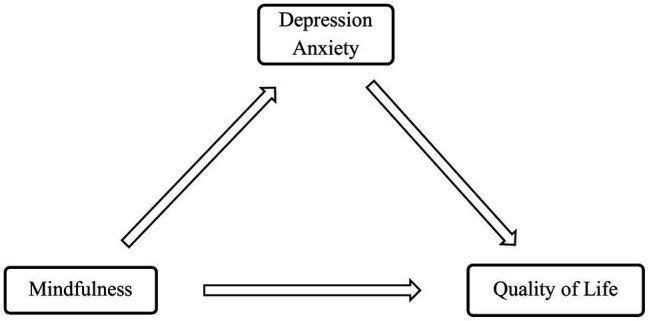
Model summary.

The study was approved by the ethical committee of the Charité – Universitätsmedizin Berlin (EA4/225/19). The present paper illustrates a cross-sectional study, which assessed the association between mindfulness, depression and anxiety, and QoL in patients with SSD. An overview of the model can be seen in Figure 1. All clinical measures and demographic information were assessed at one time point. As some participants were recruited at the outpatient and some at the inpatient ward, there was no particular time point at which the recruitment took place. Participants were approached by the researcher and provided with the study information. They did not receive monetary compensation. Informed consent to participate in the study was given in written form. After completion of the paper-pencil questionnaires, all data were pseudonymously and securely stored and processed.

### Participants

Participants were recruited in a period from November 2018 until October 2020 at the out- and inpatient ward of the Charité – Universitätsmedizin Berlin, Campus Benjamin Franklin. Due to the COVID-19 pandemic in 2020, no new participants could be recruited for the majority of the year. Of the total *n*=83 participants, *n*=54 were recruited at the in- and *n*=29 were recruited at the outpatient ward. An overview of sociodemographic variables can be seen in Table 1.

**Table 1:** Sociodemographics.

Variable	*n* | mean (*SD*)/range
Age	41.81 (13.17)/20–71
Nationality	
German	74
Turkish	5
Other than the above	4
Gender
Female	40
Male	43
Non-binary	–
Duration of illness in years	13.9 (11.64)/0–43

*SD*, standard deviation.

Eligible participants were identified and invited to participate in the study by the researcher. Inclusion criteria were (1) current treatment at the in- or outpatient facility, (2) a diagnosis from the schizophrenia spectrum according to ICD-10 code F2 given by a licensed psychiatrist prior to the eligibility assessment, and (3) being able to provide informed consent, and fluency in German language. An exclusion criterion was (a) current substance use besides nicotine or prescribed psychotropic drugs.

### Measures

All participants completed five self-rating questionnaires in German language. To assess mindfulness, three different measurements were used. They all cover different mindfulness dimensions and, therefore, provide unique information on the participants’ mindful attitude. Firstly, the one-dimensional Southampton Mindfulness Questionnaire (SMQ; Böge et al., 2020b) was administered. It refers to the past seven days and conceptualizes mindfulness as four-related aspects: (1) decentred awareness, (2) letting go, (3) non-judgment, and (4) non-aversion. The SMQ comprises 16 items that are rated on a seven-point Likert-scale ranging from (6) “*agree totally*” to (0) “*disagree totally*,” whereby (3) provides the option to answer “*unsure*”; an option that the other two questionnaires do not provide (Chadwick et al., 2008). Consequently, the total score ranges from 0 to 96, with a higher score indicating higher mindfulness. The internal consistency of the German version of the SMQ was good; with a Cronbach’s *α*=0.89 (Böge et al., 2020b), while for the current study, we found a moderate Cronbach’s *α=* 0.69.

Secondly, the Comprehensive Inventory of Mindfulness Experience (CHIME), comprising eight subscales of mindfulness, was administered. The eight subscales include (1) awareness of internal experiences, (2) awareness of external experiences, (3) acting with awareness, (4) accepting non-judgmental attitude, (5) nonreactive decentring, (6) openness to experience, (7) awareness of thoughts’ relativity, and (8) insightful understanding (Medvedev et al., 2018). The 37 items referring to the past seven days are scored on a six-point Likert-scale ranging from (1) “*almost never*” to (6) “*almost always*.” The total score ranges from 37 to 222, with a higher score indicating higher mindfulness. The CHIME showed satisfactory reliability for the total scale and the subscales of *α*>0.70 (Bergomi et al., 2012). For the current study, the reliability was *α*=0.84.

Third, a shortened version of the Freiburger Mindfulness Inventory (FMI), a 14-item one-dimensional questionnaire, was employed. Items assess participants’ mindful experiences of the past 7days and are rated on a four-point Likert-scale ranging from (1) “*rarely*” to (4) “*almost always*,” resulting in a total score from 14 to 56. Psychometrics show a high internal consistency score of *α*=0.84 (Walach et al., 2006). The current study showed an α of 0.82.

To measure clinical outcomes and symptom severity, the 21-item Depression, Anxiety, and Stress Scale (DASS-21) was employed, assessed on a four-point Likert-scale ranging from (0) “*did not apply to me at all over the last week*” to (3) “*applied to me very much last week*.” It shows internal consistencies of *α*>0.80 across the three subscales and has shown to be a useful measurement tool for patients with SSD (Page et al., 2007; Samson and Mallindine, 2014). The current study showed an α of 0.91.

To measure QoL, the short version of the World Health Organization QoL questionnaire (WHOQOL-BREF) was employed (Harper et al., 1998). It entails four domains linked to QoL: (1) physical health, (2) psychological, (3) social relationships, and (4) environment. It comprises 26 items, self-rated on a five-point Likert-scale with answers ranging from 1 to 5, indicating *how much, how complete, how often, how good,* or *how satisfied* the individual felt in the previous two weeks. High scores indicate high QoL with regard to the corresponding domain. The internal consistencies were found to be *α*=0.68 for social relationships and for the remaining domains *α*>0.80 (Skevington et al., 2004). For the current study, *α* was 0.61 for physical health, 0.74 for psychological QoL, 0.68 for social relationships, and 0.69 for environment.

### Data Analysis

All data analyses were conducted using IBM SPSS Statistics 26 for Windows 10. At first, a correlation analysis was conducted to check which variables are significantly related. Based on these correlations, subdomains of the DASS-21 and the WHOQOL-BREF significantly correlated to mindfulness instruments were included in further analyses. Multicollinearity was assessed with the variance inflation factor. In case the variance inflation factor was below 10 for the included factor, no multicollinearity was assumed. Subsequently, to assess mediation, bootstrapping was conducted using the SPSS PROCESS macro written by Hayes (2017). Bootstrapping was applied, as it was found to be among the most powerful statistical approaches to examine mediation (Hayes, 2017). Afterward, a 95% confidence interval around the indirect effect of each included variable was created and if this interval did not contain zero, a significant indirect effect was assumed. The reported coefficients are unstandardized; however, partially standardized direct effects of the independent variable on the dependent variable were reported as an indicator of the strength of the relationship in accordance with guidelines by Hayes (2017). A significance level of *α*=0.05 was applied.

## Results

For further context, the sample of the current study was compared to samples in similar studies. However, no significance can be concluded as no statistical analyses have been conducted regarding these comparisons.

Compared to the mean of another sample of individuals diagnosed with SSD, the mean SMQ score in the current study was higher (Chadwick et al., 2008). Furthermore, the mean DASS-21 score in the current study was, according to the manual of the DASS-21 (Lovibond and Lovibond, 1995): normal (0–9 points) for Depression, normal (0–7 points) for Anxiety, and normal (0–14 points) for the Stress subscale. Regarding QoL, when comparing the scores from the current study to a recent study with long-term hospitalized patients (Deenik et al., 2017), participants from the current sample on average scored slightly lower on the physical domain, the psychological domain, the social domain, and the environmental domain. See Table 2 for an overview.

**Table 2:** Descriptives of the sample.

Variable	Means in current study; *M* (*SD*)	Comparable study population; *M* (*SD*)
SMQ	46.26 (11.37)	37.15 (15.87)
DASS-21 – Depression	8.30 (5.55)	–
DASS-21 – Anxiety	6.94 (4.74)	–
DASS-21 – Stress	8.88 (5.14)	–
QoL – Physical health	12.63 (2.36)	13.6 (2.7)
QoL – Psychological	12.44 (3.1)	13.1 (2.1)
QoL – Social relationships	12.6 (3.51)	13.1 (2.9)
QoL – Environment	14.03 (2.39)	14.9 (2.1)

*M*, mean; *SD*, standard deviation; SMQ, Southampton Mindfulness Questionnaire, DASS-21, Depression, Anxiety, Stress Scale; and QoL, World Health Organization Quality of Life questionnaire.

### Correlation Analysis

Correlations between the mindfulness questionnaires SMQ and CHIME with the three domains of the DASS-21 showed a significant, negative direction. The FMI displayed a significant negative correlation with depression only. Furthermore, regarding QoL, all three mindfulness instruments correlated significantly with increased physical health and psychological QoL. Additionally, CHIME and FMI positively correlated with higher environmental QoL. For a more detailed overview of these results, see Table 3. When examining the subscales of the DASS-21 and the domains of QoL, depression had a significant negative correlation with physical health, psychological QoL, social relationships, and environment. Furthermore, anxiety had negative significant correlations with physical health, psychological QoL, and environment. Lastly, negative correlations between stress and physical health and psychological QoL were significant. An overview of these correlations can be seen in Table 4.

**Table 3:** Correlations of mindfulness with depression anxiety, stress, and quality of life.

	Depression^1^	Anxiety^1^	Stress^1^	Physical Health^2^	Psychological^2^	Social Relationships^2^	Environment^2^
**SMQ**	−0.302^**^	−0.261^*^	−0.363^**^	**0.240** ^*^	**0.262** ^*^	0.136	0.184
Mindful Observation	−0.266^*^	−0.201	−0.281^*^	0.203	**0.277** ^*^	0.019	0.093
Letting Go	−0.140	−0.133	−0.157	0.139	0.172	0.070	0.189
Absence of Aversion	−0.292^**^	−0.206	−0.283^**^	**0.217** ^*^	0.196	0.178	0.157
Non-Judgment	−0.141	−0.201	−0.314^*^	0.109	0.083	0.113	0.075
**CHIME**	−0.424^**^	−0.313^**^	−0.274^*^	**0.302** ^**^	**0.488** ^**^	0.079	**0.288** ^**^
Awareness of Internal Experiences	−0.115	0.086	0.019	0.067	0.211	−0.003	**0.222** ^**^
Awareness of External Experiences	−0.146	−0.098	−0.084	−0.010	0.160	−0.019	0.062
Acting with Awareness	−0.338^**^	−0.411^*^	−0.544^**^	**0.269** ^*^	**0.327** ^**^	0.033	0.118
Accepting Non-Judgmental Attitude	−0.536^**^	−0.494^**^	−0.436^**^	**0.461** ^**^	**0.477** ^**^	0.209	**0.220** ^*^
Nonreactive Decentring	−0.347^**^	−0.263^*^	−0.245^*^	**0.220** ^*^	**0.472** ^**^	0.114	**0.228** ^*^
Openness to Experience	−0.110	−0.094	−0.081	0.069	−0.018	0.004	−0.077
Awareness of Thoughts’ Relativity	−0.060	−0.043	0.136	0.136	**0.226** ^*^	−0.083	0.153
Insightful Understanding	−0.147	−0.027	0.079	0.070	0.184	0.034	**0.260** ^*^
**FMI**	−0.288^**^	−0.175	−0.189	**0.315** ^**^	**0.413** ^**^	0.106	**0.281** ^**^

1 assessed by the DASS-21.

2 assessed by the WHOQOL-BREF.

* *p<* 0.05;

** *p<* 0.001.

**Table 4:** Correlations of depression, anxiety, and stress with quality of life.

	Physical Health^2^	Psychological^2^	Social Relationships^2^	Environment^2^
Depression^1^	**−0.567** ^**^	**−0.651** ^**^	**−0.234** ^*^	**−0.307** ^**^
Anxiety^1^	**−0.451** ^**^	**−0.384** ^**^	−0.020	**−0.222** ^*^
Stress^1^	**−0.305** ^**^	**−0.332** ^**^	0.002	−0.102

1 assessed by the DASS-21.

2 assessed by the WHOQOL-BREF.

* *p<* 0.05;

** *p<* 0.01.

### Process Analysis

All assumptions for linear regression were met (Hayes, 2017). As covariates, age, gender, duration since the first onset of the disorder, and recruitment site (in- or outpatient ward) have been examined. The recruitment site was found to significantly predict the “physical health,” “psychological,” and “environmental” domains of QoL. Therefore, it was included as a covariate in all PROCESS models predicting physical health. The exact results can be seen in Table 5. Participants at the inpatient ward indicated slightly higher DASS-21 scores in the subdomains depression (outpatients: *M*=7.10, *SD*=5.67; inpatients: *M*=8.94, *SD*=5.54) and stress (outpatients: *M*=7.69, *SD*=5.05; inpatients: *M*=9.52, *SD*=5.12) and a significantly higher score in the anxiety domain (outpatients: *M*=5.21, *SD*=3.92; inpatients: *M*=7.87, *SD*=4.91); *t*=−2.52, *p<* 0.05.

**Table 5:** Potential covariates and their effects on quality of life domains.

	Physical Health^1^		Psychological^1^		Social Relationships^1^		Environment^1^	
	*t*	*p*	*t*	*p*	*t*	*p*	*t*	*p*
Age	−0.661	0.511	−0.235	0.815	0.251	0.831	−0.705	0.483
Gender	0.601	0.550	0.137	0.892	0.840	0.404	−0.073	0.942
Duration of illness	0.023	0.981	−1.133	0.261	0.497	0.621	−0.098	0.922
Recruitment site	**−4.288**	**0.001**	**−2.258**	**0.027**	−1.131	0.194	**−2.659**	**0.009**

1 assessed by the WHOQOL-BREF.

**Figure 2:**
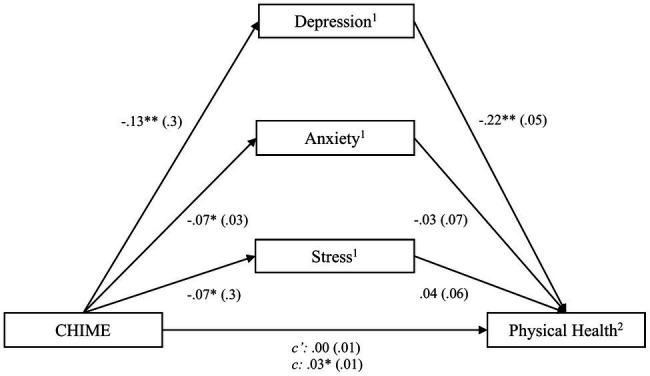
Process outcomes including CHIME and physical health. ^*^ *p*<0.05; ^**^ *p*<0.01, ^1^assessed by the DASS-21, ^2^assessed by the WHOQOL-BREF. For simplicity, the covariate, recruitment site, is not included in this and the following figures, although being included in the analysis. For more detailed results, see Table 5.

After controlling for recruitment site as a covariate, the SMQ score was not found to predict the QoL domain physical health. Therefore, the mediation effect was not examined. When including the CHIME instead of the SMQ, the model [*F*(5,77)=12.56, *p*<0.001] explained *R*^2^ *=* 0.45 of the variance of physical health. The CHIME score was found to predict physical health [*b*=0.03, *SE*=0.01, *t*(80)=2.46, *CI* (0.01; 0.05)] and all three subdomains of the DASS-21 – depression [*b*=−0.12, *SE*=0.03, *t*(80)=−4.02, *CI* (−0.17; −0.06)], anxiety [*b*=−0.07, *SE*=0.03, *t*(80)=−2.67, *CI* (−0.12; −0.02)], and stress [*b*=−0.07, *SE*=0.03, *t*(80)=−2.36, *CI* (−0.12; −0.01)]. Combined into one linear regression model, the CHIME no longer predicted physical health, however depression did, indicating that depression mediated the relationship between CHIME score and physical health (see Figure 2). Using the FMI, only the DASS-21 subscale depression [*b*=−0.20, *SE*=0.08, *t*(80)=−2.41, *CI* (−0.37; −0.36)] was predicted by mindfulness as well as *physical health* [*b*=0.07, *SE*=0.03, *t*(80)=2.23, *CI* (0.01, 0.14)]. The model was significant, *F*(3,79)=21.40, *p*<0.01, and it explained *R*^2^ *=* 0.45 of the variance. The partially standardized direct effect of the FMI score on *physical health* was c′_ps_=0.01, indicating that two subjects who differ one unit in mindfulness differ by about 1% of a standard deviation in their physical health.

**Figure 3:**
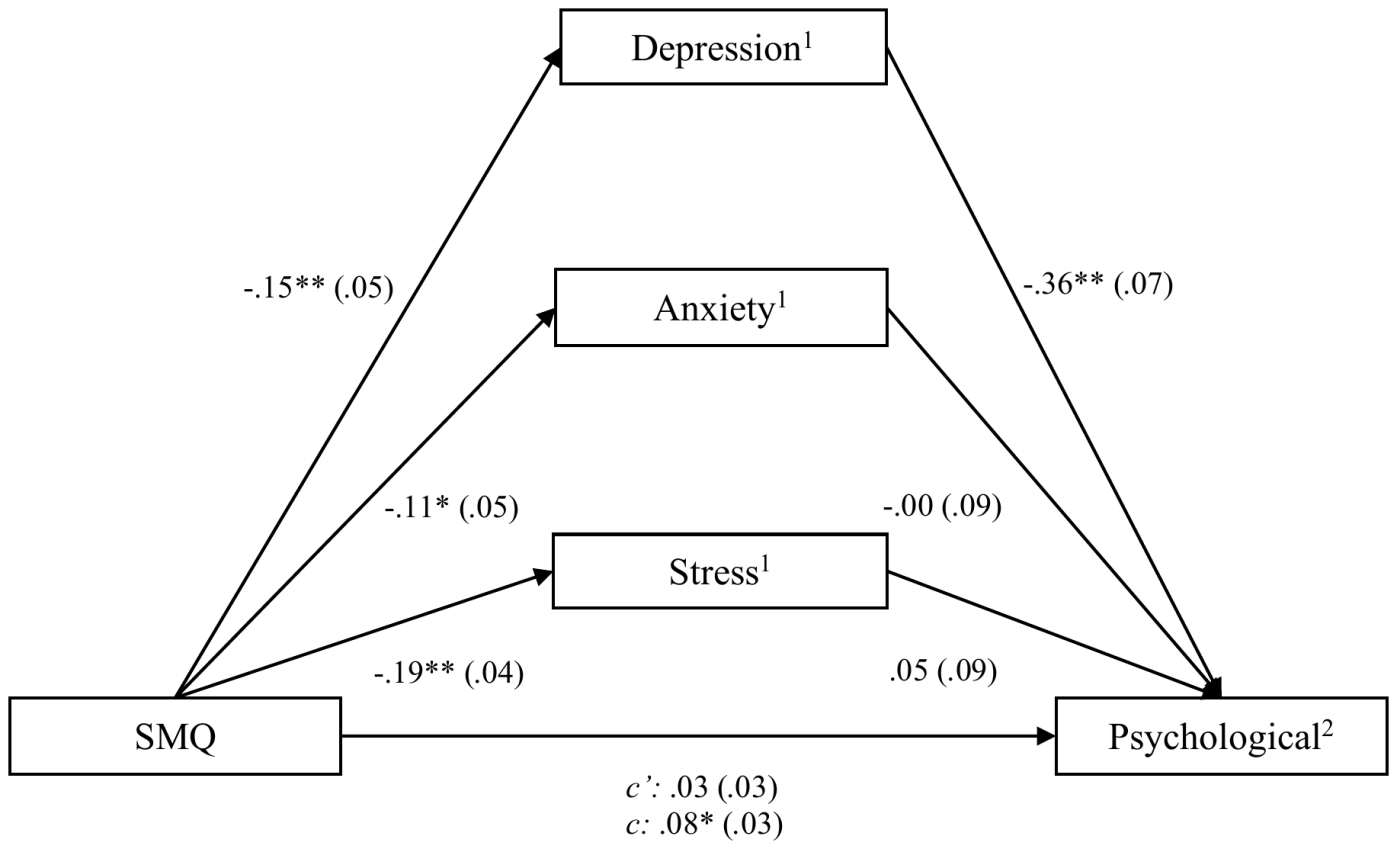
Process outcomes including SMQ and psychological quality of life. ^*^ *p*<0.05; ^**^ *p*<0.01, ^1^assessed by the DASS, ^2^assessed by the WHOQOL-BREF.

Regarding the psychological QoL subdomain, results indicated that the SMQ score predicted all three subdomains of the DASS-21 – depression [*b*=−0.14, *SE*=0.05, *t*(80)=−2.63, *CI* (−0.24; −0.03)], anxiety [*b*=−0.09, *SE*=0.04, *t*(80)=−2.07, *CI* (−0.18; −0.01)] and stress [*b*=−0.16, *SE*=0.05, *t*(80)=−3.27, *CI* (−0.25; −0.06)], and psychological QoL [*b*=0.06, *SE*=0.03, *t*(80)=2.10, *CI* (0.01; 0.12)]. When adding the DASS-21 subdomains into the model predicting psychological QoL, only depression remained a significant predictor, indicating a mediation. The linear regression model was significant, *F*(5,77)=12.63, *p*<0.01, and it explained *R*^2^ *=* 0.45 of the variance (see Figure 3). The partially standardized direct effect of the SMQ score on psychological QoL was c′_ps_=0.01. Results regarding the CHIME as the instrument for assessing mindfulness indicated an effect of mindfulness on psychological QoL [*b*=0.07, *SE*=0.02, *t*(80)=4.77, *CI* (0.04; 0.10)]. This significant effect remained when including the DASS-21 subscales in the regression model. Therefore, mediation was given. The model was significant, *F*(5,77)=15.28, *p*<0.01, and it explained *R*^2^ *=* 0.50 of the variance. The partially standardized direct effect of the CHIME score on psychological QoL was c′_ps_=0.01. As the third employed mindfulness questionnaire, the FMI score was a significant predictor of psychological QoL [*b*=0.16, *SE*=0.05, *t*(80) =3.63, *CI* (0.07; 0.25)]. After including depression into the regression model, both variables were significant, supporting a mediation effect. The model was significant, *F*(3,79)=25.20, *p*<0.01, and it explained *R*^2^ *=* 0.49 of the variance. The partially standardized direct effect of the FMI score on psychological QoL was c′_ps_=0.03.

**Figure 4:**
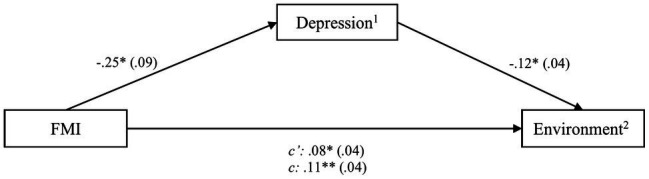
Process outcomes including FMI and environmental quality of life. ^*^ *p*<0.05; ^**^ *p*<0.01, ^1^assessed by the DASS, ^2^assessed by the WHOQOL-BREF.

Lastly, environmental QoL was assessed with regard to its association with the CHIME and the FMI. The CHIME was found to significantly predict the environment domain [*b*=0.03, *SE*=0.01, *t*(80)=2.39, *CI* (0.01; 0.06)]. When controlling for DASS-21 scores, the relationship became insignificant and neither depression, anxiety, nor stress scores showed significant associations, which contradicted a mediation. With regard to the FMI, the initial significant association between mindfulness and environment [*b*=0.08, *SE*=0.04, *t*(80)=2.11, *CI* (0.01; 0.15)] was weakened after adding depression, but both were still significant when added into one regression model, indicating mediation. The model including FMI score and depression was significant, *F*(3,79)=5.60, *p*<0.01, and explained *R*^2^ *=* 0.18 of the variance of environment (see Figure 4). The partially standardized direct effect of the FMI score on environment QoL was c′_ps_=0.02.

## Discussion

The current study aimed at examining the mechanisms of mindfulness and the influence of depressive and anxiety symptoms on the effect of mindfulness on QoL in individuals with SSD. The participants included in the current study were, in comparison with similar studies (Lovibond and Lovibond, 1995; Chadwick et al., 2008; Deenik et al., 2017), more mindful, reported less QoL on all domains, and scored within the *normal* range of depressive and anxiety symptoms, as defined by the authors of the DASS-21, indicating good generalizability of the results.

Initially, it was hypothesized that high levels of mindfulness are associated with high QoL as well as with low levels of depression and anxiety, and furthermore, that depression and anxiety mediate the relationship between mindfulness and QoL. Concerning the first two hypotheses, both correlational analysis and regression analysis indicated an association between high levels of mindfulness and low levels of depression and anxiety, assessed by the DASS-21 subdomains anxiety and stress (Huppert and Smith, 2005). Additionally, a relation between high levels of mindfulness and high scores on the QoL domains was shown, which is in line with previous research (López-Navarro et al., 2015). All the three mindfulness assessment tools were found to be weakly to moderately positively associated with psychological QoL and physical health, and two (CHIME and FMI) with environmental QoL. Social relationships, the remaining domain of QoL as assessed by the WHOQOL-BREF, were not found to be related to mindfulness.

Concerning the third hypothesis, mediation analysis revealed that the association between mindfulness and QoL is mediated by lower levels of depressive and anxiety symptoms. When comparing the two mediators, depressive symptoms were found to explain a higher proportion of variance than anxiety. The aspects of mindfulness most strongly associated with depression and anxiety appear to be *Acting with awareness, Non-judgment,* and *Nonreactive decentring*. This is in line with findings of a recent meta-analysis (Carpenter et al., 2019), which examined the relationship between mindfulness facets and affective symptoms. Therefore, these mindful mechanisms might play crucial roles in the process-oriented approach toward SSD, being identified as the counterpart to rumination, a key aspect in psychopathology in general and SSD in specific (Ludwig et al., 2020). Due to increased self-insight, individuals can become aware of inner processes and achieve an active sense of inner experiences. Thereby, individuals can learn to refrain from judging their symptoms and ultimately, differentiate between their experiences and their self-concept (Böge et al., 2020a). By taking this active metacognitive stance toward their symptoms (Böge et al., 2020a; Jansen et al., 2020), emotional and cognitive reactivity toward negative experiences are reduced (Jansen et al., 2020) and focus can shift toward more pleasant, disorder-unrelated experiences. This cognitive approach can be linked back to basic principles of cognitive therapy, proposing that not the experience, but the cognitive *reaction* toward the event determines to what extent the individual is affected (Beck, 1979). By reclaiming agency over their own symptoms, a sense of self-agency can enhance the subjectively experienced psychological QoL. Besides the psychological effects of MBIs, the impact on other health-related domains, such as physical health, is less well established (Greeson et al., 2018). Some of the symptoms characterizing depression include loss of energy and deprived sleep (American Psychiatric Association, 2013). As proposed by research on stress-related disorders, mindfulness-based stress reduction can positively impact sleep quality and elevate energy levels (Greeson et al., 2018). Consequently, by experiencing fewer disorder-related symptoms, fewer aspects of physical health are affected. The remaining domain of QoL, environmental QoL, seems to be associated with mindfulness and mediated by depressive symptoms. One could argue that by being more mindful, individuals are more accepting of their circumstances and, therefore, feel less impaired by them.

The outcomes of this study must be viewed with caution, as the study design contains limitations. First, a cross-sectional design was implemented and therefore, no conclusions can be drawn regarding causality or the direction of the relationship between the employed variables. Future research needs to employ a randomized-controlled design to investigate the effects of a mindfulness intervention on the variables included in this study to draw conclusions about causality. Moreover, in the current study, patients at the inpatient ward were found to have slightly higher DASS-21 scores in the domains Depression and Stress and a significantly higher score in the Anxiety domain. In the current study, the statistical design included recruitment site as a covariate in all models in order to address potential confounding. However, the severity of positive and negative symptoms of SSD was not assessed in this study and should be addressed in future projects. Therefore, it cannot be ruled out that symptom severity might play an important role in the examined relationships. Also, there was no assessment of current medication regime. Prescribed psychotropic medication could have had an impact on internal processes investigated in this study and therefore should be systematically assessed in future trials. Furthermore, as the study employed exclusively self-rated questionnaires, more objective means of assessing the included variables should be employed in future projects. Lastly, the study contained a rather small sample and future trials should include a larger sample to support the findings of the current study.

Additionally, this study’s statistical model assumed linear relationships in line with causal steps model of Baron and Kenny (1986). However, psychological processes can hardly be conceptualized in linear models assuming a unidirectional way (Hofmann et al., 2020). In other words, it might be that more mindful individuals experience less depression or anxiety, but vice versa, individuals lower in depression or anxiety may have more capacity to engage in mindfulness. Therefore, this approach is limited and cannot capture the complex nature of the underlying processes fully. Future studies investigating the mechanisms of MBIs should overcome these methodological shortcomings and acknowledge the interrelatedness of variables and their dynamic network (Hofmann et al., 2020). For instance, by employing a dynamic network approach, psychopathology’s complexity can be recognized by displaying the multidirectional paths in patterns that highlight the central symptoms (Borsboom and Cramer, 2013). This approach would be in line with the recent argumentation that process-based interventions, which focus on change processes and a more ideographic treatment of patients (Hayes et al., 2019), could be the future of psychotherapy (Hofmann et al., 2020).

Nevertheless, the current study provides initial evidence on the mechanisms of mindfulness. The results once more underline the transdiagnostic relevance of mindfulness (Greeson et al., 2018), as while the target group included individuals with SSD, lowered depressive and anxiety symptoms were found to be associated with increased mindfulness. Studies like this might contribute to preparing the ground for a possible paradigm shift – from a narrow perspective of viewing mental disorders as distinct categories toward focusing on the common elements shared across disorders (Borsboom, 2017). By following transdiagnostic approaches, researchers can increasingly treat symptoms as dimensional variables that are not unique to one disorder but are central elements in several psychopathological states and are therefore responsible for comorbidities (Zhou et al., 2019). Consequently, in clinical settings, transdiagnostic interventions that target specific symptoms, such as MBIs, improve features of one disorder and comorbid, co-occurring disorders (Bullis et al., 2019). Thereby, these interventions can be employed across disorders, including patients with a variety of symptoms and diagnoses. Additionally to SSD, a recent systematic review has further indicated the clinical interest of MBIs for individuals with first episode psychosis and with ultra-high risk for transition to psychosis, highlighting similar underlying mechanisms of action, such as reduced anxiety and improved QoL (Vignaud et al., 2019). MBIs might therefore also be employed as early interventions preventing in prodromal phases of SSD.

In conclusion, in line with the hypotheses, mindfulness was found to be negatively related to anxiety and depression and positively to QoL in individuals with SSD. Furthermore, as hypothesized, depression and anxiety are found to mediate the relationship between mindfulness and QoL, whereby lower depressive and anxiety symptoms are associated with higher reported QoL in several domains. Initial evidence for the transdiagnostic and process-based clinical relevance of MBIs for SSD has been found and future studied can elaborate on these results.

## Data Availability Statement

The raw data supporting the conclusions of this article will be made available by the authors, without undue reservation.

## Ethics Statement

The studies involving human participants were reviewed and approved by Charité’s Ethics Committee. The patients/participants provided their written informed consent to participate in this study. Informed consent was obtained from all individual participants included in the study. Patients signed informed consent regarding anonymously publishing their data.

## Author Contributions

NB designed and executed the study, conducted the data analyses, and wrote the paper. EH, MZ, and TT collaborated with the design and editing of the final manuscript. IH collaborated with the design and execution of the study and editing of the manuscript. MB edited the final manuscript. GP assisted with the study design and edited the manuscript. KB assisted in the study design and editing of the manuscript. All authors contributed to the article and approved the submitted version.

## Conflict of Interest

The authors declare that the research was conducted in the absence of any commercial or financial relationships that could be construed as a potential conflict of interest.

## Publisher’s Note

All claims expressed in this article are solely those of the authors and do not necessarily represent those of their affiliated organizations, or those of the publisher, the editors and the reviewers. Any product that may be evaluated in this article, or claim that may be made by its manufacturer, is not guaranteed or endorsed by the publisher.
